# Nitrogen transport and assimilation in tea plant (*Camellia sinensis*): a review

**DOI:** 10.3389/fpls.2023.1249202

**Published:** 2023-09-22

**Authors:** Wenjing Zhang, Kang Ni, Lizhi Long, Jianyun Ruan

**Affiliations:** ^1^ Key Laboratory of Tea Plant Biology and Resources Utilization, Ministry of Agriculture, Tea Research Institute, Chinese Academy of Agricultural Sciences, Hangzhou, China; ^2^ Graduate School of Chinese Academy of Agricultural Sciences, Beijing, China; ^3^ Xihu National Agricultural Experimental Station for Soil Quality, Hangzhou, China

**Keywords:** nitrogen transport, nitrate reduction, ammonia assimilation, NUE, camellia sinensis, challenges and prospects

## Abstract

Nitrogen is one of the most important nutrients for tea plants, as it contributes significantly to tea yield and serves as the component of amino acids, which in turn affects the quality of tea produced. To achieve higher yields, excessive amounts of N fertilizers mainly in the form of urea have been applied in tea plantations where N fertilizer is prone to convert to nitrate and be lost by leaching in the acid soils. This usually results in elevated costs and environmental pollution. A comprehensive understanding of N metabolism in tea plants and the underlying mechanisms is necessary to identify the key regulators, characterize the functional phenotypes, and finally improve nitrogen use efficiency (NUE). Tea plants absorb and utilize ammonium as the preferred N source, thus a large amount of nitrate remains activated in soils. The improvement of nitrate utilization by tea plants is going to be an alternative aspect for NUE with great potentiality. In the process of N assimilation, nitrate is reduced to ammonium and subsequently derived to the GS-GOGAT pathway, involving the participation of nitrate reductase (NR), nitrite reductase (NiR), glutamine synthetase (GS), glutamate synthase (GOGAT), and glutamate dehydrogenase (GDH). Additionally, theanine, a unique amino acid responsible for umami taste, is biosynthesized by the catalysis of theanine synthetase (TS). In this review, we summarize what is known about the regulation and functioning of the enzymes and transporters implicated in N acquisition and metabolism in tea plants and the current methods for assessing NUE in this species. The challenges and prospects to expand our knowledge on N metabolism and related molecular mechanisms in tea plants which could be a model for woody perennial plant used for vegetative harvest are also discussed to provide the theoretical basis for future research to assess NUE traits more precisely among the vast germplasm resources, thus achieving NUE improvement.

## 1 Introduction

Nitrogen is an essential mineral nutrient for plant growth and reproduction. Apart from being a fundamental building block of proteins and nucleic acids, N also participates in carbon fixation through photosynthesis as a component of chlorophyll (Bernard and Habash, 2009). In agricultural production, applying N fertilizers generally leads to significant yield increases (Suárez et al., 2002; Liu et al., 2021c), for which N fertilizers’ use is expected to increase up to 236 million metric tons to meet the global food demands by 2050 (Beatty and Good, 2018). However, less than 50% of the applied N as fertilizer is absorbed by plants and harvested in grains (Raun and Johnson, 1999; Camargo et al., 2005). Thus, a high amount of “unuse” N supplied as fertilizer is transferred to water and the atmosphere, resulting in energy waste, soil acidification, water eutrophication and greenhouse gas emissions (Godfray et al., 2010; Liu et al., 2010). This negative environmental consequence of nitrogen fertilization became a huge challenge for stable and sustainable agricultural production (Bodirsky et al., 2014). There is an urgent need for research advances on N metabolism in the ecosystem; in this context, we need to improve N use efficiency (NUE) by crops, for which the genetic potential for N uptake and assimilation must be further explored.

Tea is processed from the leaves of *Camellia sinensis* (L.) O. Kuntze and becoming one of the most widely non-alcoholic beverages consumed worldwide due to its unique taste and potential health benefits (Wei et al., 2018). Since 2011, the global planting area of tea have increased steadily and gradually, from 3.84 million hectares in 2011 to 5.09 million hectares in 2020 (Liu et al., 2023). This perennial evergreen woody plant is cultivated in over 30 countries, and China has the greatest cultivated area (Zhang et al., 2019b; Lei et al., 2022). In 2022, tea planting area of China reached 3.33 million hectares (Mei and Zhang, 2022). The geographic origin of the tea plant is assigned to Yunnan province and neighboring regions in southwestern China (Chen et al., 2005). China has traditionally been the largest tea producer worldwide with abundant germplasm resources, and China’s tea have been exported to more than 140 countries or regions (Wei et al., 2012). Currently, many cultivated tea varieties are extensively grown in tropical and subtropical regions across the world, and tea cultivation may increase the local smallholder income, especially in mountainous areas, contributing to local economic development (Yao et al., 2012). The N concentration in young buds and leaves is about 60-70 g·kg^−1^ (Ma et al., 2013). Tea plants form new shoots every season, and multiple picking and pruning have been done. In agricultural production, tea plants have a high demand for N, which is generally fulfilled through fertilization, active N uptake, assimilation and translocation, as well as remobilization processes. In China, the average annual N inputs reach 300-450 kg·hm^−2^ to cover tea N demand; an excessive N application rate has been reported in over 30% of the tea plantation area (Ma et al., 2013; Ni et al., 2019). These numerical data reinforce the crucial and urgent need for optimizing the NUE of tea plant. A series of interconnected processes, including N transport, assimilation and remobilization, are involved in NUE, thus the understanding on N metabolism at molecular level will provide the basis for a more rational application of N fertilizers during tea production.

**Figure 1:**
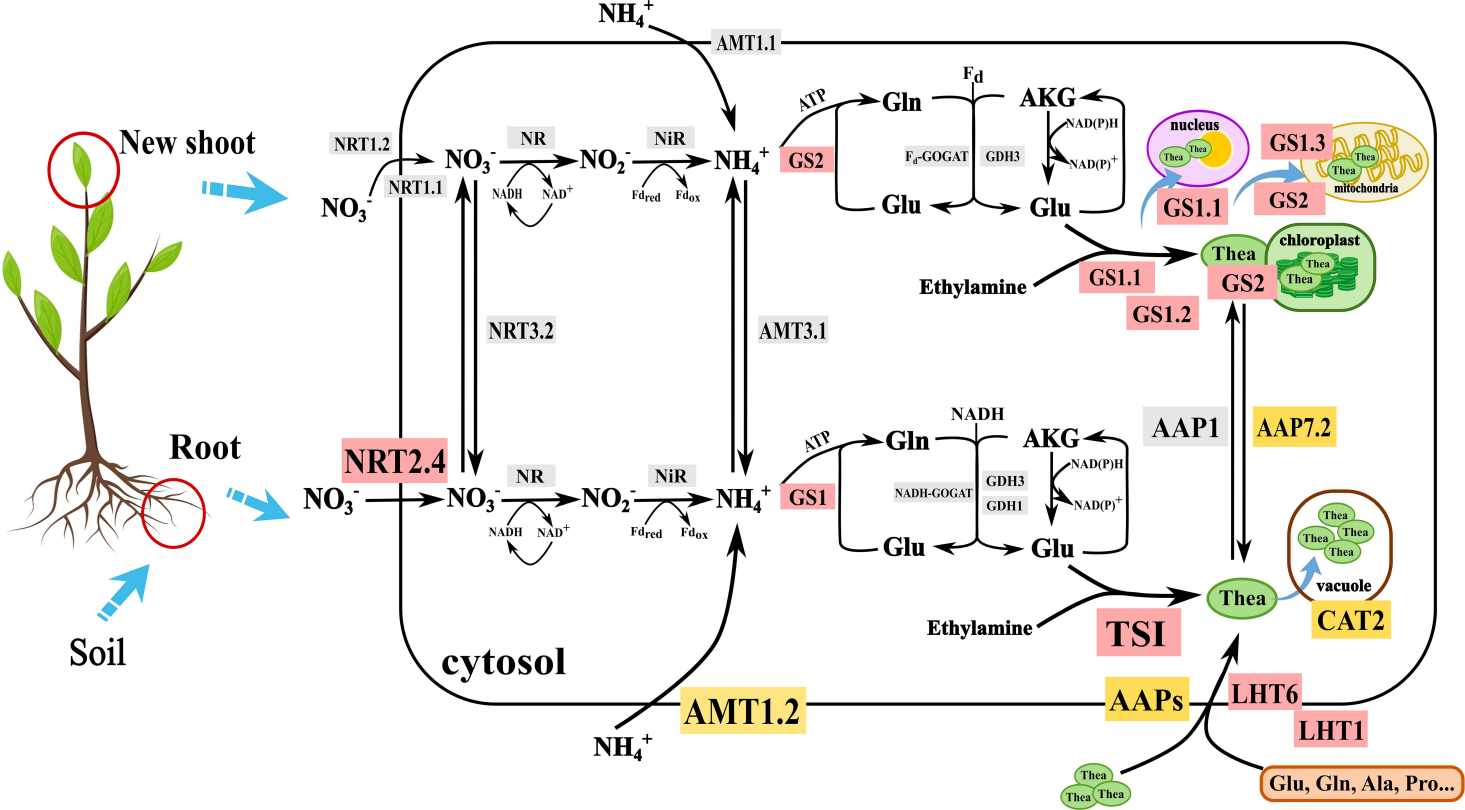
Molecular mechanism of nitrogen nutrient absorption and utilization in tea plant. NRT, nitrate transporter; NR, Nitrate reductase; NiR, Nitrite reductase; AMT, Ammonium transporter; GS, Glutamine synthetase; GOGAT, Glutamate synthase; GDH, Glutamate dehydrogenase; Gln, Glutamine; Glu, Glutamic acid; TS, Theanine synthetase; Thea, Theanine; LHT, Lysine and histidine transporter; CAT, Cationic amino acid transporter; Ala, Alanine; Pro: Proline; AKG, α-Ketoglutaric acid. Gray background represents the genes just were cloned in vitro; Yellow background represents functions of these proteins were validated in yeast; The red background represents functions of these proteins were validated in *Arabidopsis*, *Nicotiana tabacum* or *Camellia sinensis*.

Nitrogen is involved in many important metabolic pathways closely related to the synthesis of amino acids (AAs), caffeine, polyphenols, and other substances responsible for tea quality (Tang et al., 2020). Inorganic N sources, including ammonium (NH_4_^+^) and nitrate (NO_3_^-^), and small organic N-containing compounds can be uptaken from the soil by the tea plant roots and subsequently transported to the leaves by ammonium transporters (AMTs), nitrate transporters (NRTs), and amino acid transporters (AATs). The absorbed NO_3_^−^ is first reduced into nitrite (NO_2_^−^) in the cytoplasm by nitrate reductase (NR) and further reduced to NH_4_^+^ in plastids by nitrite reductase (NiR). Ammonium assimilation involves the conversion of inorganic N to organic N, mainly through the glutamine-glutamate (GS-GOGAT) cycle, catalyzed by glutamine synthetase (GS) and glutamine-2-oxoglutarate aminotransferase/ glutamate synthase (GOGAT) (Bernard and Habash, 2009; Liu et al., 2022). It is noteworthy that glutamate and ethylamine are catalyzed by theanine synthetase (TS) to biosynthesize theanine (γ-glutamyl-L-ethylamide), a unique non-proteinogenic amino acid responsible for umami taste and healthy beneficial component in tea. Thus, the content of Thea is an important indicator for cultivar breeding and evaluating NUE. These processes are schematically illustrated in **Figure 1**. Further details on substrates, transporters, enzyme isoforms, and cell compartments relevant to the N cycle in tea plants are given in the following sections.

Since the tea plant genome was sequenced (Xia et al., 2020), many enzymes involved in N metabolism and their encoding genes were identified. Nitrogen dynamic regulation and physiological function were widely investigated in tea plant, as these are all critical aspects to improve NUE. In this article, we outline the results of recent investigations about the mechanisms underlying: (1) N absorption and transport in the form of NH_4_^+^, NO_3_^−^, and AAs; (2) metabolic reduction of nitrate; (3) ammonia assimilation and theanine (γ-glutamyl-L-ethylamide) biosynthesis. We also discuss the use of genetic, genomic, and phenotyping technologies for improving NUE by tea plants and stress the relevance of understanding the genetic basis of tea plant adaptive responses to different N forms.

## 2 General nitrogen utilization traits by tea plants

### 2.1 Tea plants acquire N preferentially as NH_4_^+^

Tea plant shows a preferential uptake of N in the form of ammonium (NH_4_^+^-N). Using the scanning ion-selective electrode technique, Ruan et al. (2016) found that the NH_4_^+^ influx rate in the roots of tea plant was higher than that of NO_3_^−^, and the presence of NH_4_^+^ would promote NO_3_^−^ influx rate. The yield of young shoots, total root length, N uptake rate, and the contents of caffeine, theanine (Thea), glutamine (Gln), glutamate (Glu) and aspartate (Asp) in tea leaves were significantly higher when NH_4_^+^ was the main N source, compared with NO_3_^−^ (Ruan et al., 2007; Ruan et al., 2010; Ruan et al., 2019; Wang et al., 2022a). NH_4_^+^-N can also promote phosphorus (P) uptake (Chen et al., 2019) and increase iron (Fe) and chloride (Cl) contents in mature leaves and sulfur (S) content in the roots (Tang et al., 2019). As a signaling molecule, NH_4_^+^ could induce Thea and catechin biosynthesis in a short period (Liu et al., 2017b; Huang et al., 2018). By sensing NH_4_^+^ levels, lysine-acetylated and crotonylated proteins profoundly influenced some primary metabolic processes involved in amino acid metabolism, photosynthesis, glycolysis, and carbon fixation (Jiang et al., 2018; Sun et al., 2019).

### 2.2 Nitrogen concentration influences tea plant growth and biochemical profile

To obtain an appropriate amount of harvestable product, i.e., young buds and leaves, multiple tender shoots are picked from the plants every year. Adequate N nutrition is necessary to increase the formation of young shoots, enhance the growth vigor and maintain the C/N balance (Ruan et al., 2010). A balanced C/N ratio is also important to promote chlorophyll biosynthesis (Yang, 2011) and to ensure adequate availability of free AAs (Liu et al., 2020; Wang et al., 2022a), thus providing N reserve for reproductive growth (Fan et al., 2019).

N metabolism of tea plant is dynamically regulated by environmental factors. Likewise, the growth of lateral roots was regulated by N levels: their length and numbers decreased with increasing N concentrations (Chen et al., 2023; Hu et al., 2023). Under N deficiency, the content of N, L-Thea, and chlorophyll decreased significantly. The activity of many antioxidant enzymes and leaf CO_2_ assimilation capacity also diminished (Lin et al., 2016; Lin et al., 2019). However, low N levels positively regulated the expression of phosphate transporter genes and promoted flavonoids and polyphenols synthesis in tea leaves (Lin et al., 2023b). Appropriate N supply contributes to the aroma and flavor quality of tea infusion. The activity of the rate-limiting enzyme for N assimilation, GS, increased with N application level, and the content of total AAs, alcohols, and ketone compounds conferring aroma also increased, thus promoting tea products’ integrated quality (Ruan et al., 2010; Deng et al., 2012; Liu et al., 2021b).The accumulation of caffeine, a component of the bitter taste and a central nervous system stimulant in tea, can be increased with the increasing N supply (Ruan et al., 2010). Sufficient N also promotes flavonol glycoside biosynthesis through the expression of relevant genes and the accumulation of the corresponding substrate carbohydrates (Dong et al., 2019). Lipidomic studies revealed that the content of precursors for the formation of aroma-related substances such as monogalactosyl diaclyglycerol (36:6 MGDG) and digalactosyl diacylglycerol (36:6 DGDG) increased when the N fertilizer was applied at adequate amounts, while an excessive N application led to overaccumulation of hexenol and hexenal, compounds which cause an unpleasant grassy smell in tea (Liu et al., 2017a). With the increase in N supply, more C was allocated to N-containing compounds in mature tea leaves and roots, leading to a decrease in flavonoid concentration in the young shoots (Liu et al., 2021a). Long-term N overfertilization reduced significantly benzyl alcohol and 2-phenylethanol contents in tea leaves, as well as those of (E)-nerolidol and indoles in withering leaves, becoming not conducive to the generation of floral and fruity fragrances (Chen et al., 2021).

## 3 Nitrogen transport in plants

In a wide range of organisms, N transport as NH_4_^+^, NO_3_^−^, and soluble organic compounds across membranes is mediated by transporter proteins (Wirén et al., 1997). These transporters can be divided into high-affinity transporter systems (HATS) and low-affinity transporter systems (LATS), depending on the specific substrate affinity. The external N level also regulates the affinities of transporters. For instance, there are inducible high-affinity transporter systems (iHATS) and constitutive high-affinity transporter systems (cHATS) to accomplish NO_3_^−^ transport (Crawford and Glass, 1998; Forde, 2000). These transport proteins play a vital role in both short- and long-distance translocation of N inorganic ions and N-organic compounds.

### 3.1 NH_4_^+^ transport

The membrane-localized ammonium transporter/methylammonium permease (AMT/MEP) facilitates the import and export of NH_4_^+^ (Howitt and Udvardi, 2000). In higher plants, AMT proteins can be divided into two types: AMT1 and AMT2. Most AMT1 proteins belong to the HATS group and are synergically involved in NH_4_^+^ transport through the apoplastic and the symplastic routes (Yuan et al., 2007). AMT2 plays a role in the translocation of NH_4_^+^ from roots to shoots (Giehl et al., 2017). The transcription of the gene encoding this protein is tightly controlled through multiple factors, including external N level, circadian rhythm, hormone contents, and mycorrhizal symbiosis (Couturier et al., 2007; Kobae et al., 2010; Li et al., 2012; Li et al., 2016).

To cope with elevated NH_4_^+^ concentrations, the AMT activity may be post-translationally modified via the reversible phosphorylation of the cytosolic C-terminal region, thus allowing rapid adaptation to variable environmental conditions (Yuan et al., 2013; Wu et al., 2019). In tea plants, *CsAMTs* expression seems to be tissue-specific: *CsAMT1.2* reached the highest transcript abundance in roots, while *CsAMT1.4* was mainly expressed in flower buds. However, *CsAMT1.1* and *CsAMT3.1* were highly expressed in all tissues, suggesting that these genes might have diverse functions in NH_4_^+^ transport (Zhang et al., 2018; Wang et al., 2022c; Zhang et al., 2022a). Likewise, *AMTs* expression levels are responsive to changes in NH_4_^+^ availability. In roots, *CsAMT1.1* expression peaked at 12 h after the exogenous NH_4_^+^ resupply, while *CsAMT3.1* showed an upward trend after 24 h and *CsAMT1.2* expression level increased at 10 h, with a 2.5-fold change compared to 0 h, and then decreased again by 24 h. In NH_4_^+^-treated leaves, *CsAMT1.1* expression was up-regulated only after 4 h, exhibiting a 4.75-fold increase, whereas *CsAMT1.2* and *CsAMT3.1* expression levels did not change until 24 h later. These data indicate that NH_4_^+^ transport in tea roots is mainly regulated by *CsAMT1.2*, while in leaves, the NH_4_^+^ induction is mainly controlled by *CsAMT1.1* in the short term (Tang et al., 2020). Across different experimental NH_4_^+^ concentrations, most *CsAMTs* were expressed at higher levels in leaves than roots, except for *CsAMT1.2*, *CsAMT1.4*, and *CsAMT2.1a*. Remarkably, *CsAMT1.2* expression was significantly higher in roots than leaves under NH_4_^+^ deficiency (0 mM NH_4_^+^) or at 4 mM NH_4_^+^, demonstrating the major role of this transporter in NH_4_^+^ uptake. Other genes involved in NH_4_^+^ transport, such as *CsAMT2.1b*, *CsAMT3.3*, *CsAMT4.1a*, *CsAMT4.1b*, *CsAMT4.1c*, and *CsAMT4.1d*, exhibited similar expression profiles, with a decreasing trend under low N supply and a notorious induction under high N supply (Wang et al., 2022c). Furthermore, this report indicates that *CsAMTs* expression in tea leaves is differentially regulated over time by abiotic stresses, including drought and salinity, as well as after methyl jasmonate treatments. Under these treatments, specific *CsAMTs* genes were up-regulated or down-regulated in different ways, suggesting different functions to cope with various stresses (Wang et al., 2022c).

Transcriptome data revealed that *CsAMT1.2* expression could be highly induced by NH_4_^+^-resupply; weighted gene co-expression network analyses and the functional validation in an NH_4_^+^-uptake defective yeast line further corroborated that the high-affinity transporter *CsAMT1.2* was a “hub gene” in the N metabolic network of tea plants, controlling NH_4_^+^ uptake from the soil to the roots (Zhang et al., 2020). Also, Wang et al. (Wang et al., 2022c) found that 11 yeast transformant lines grew well on 0.3 mM NH_4_^+^ as the sole N source, indicating their high affinity for NH_4_^+^ permeation. The transcriptional regulation of *CsAMTs* differed even at the cultivar level (Li et al., 2017). *CsAMT1.1* and *CsAMT1.5* expression levels were significantly higher in the roots of the FuDingDaBaiCha cultivar than Longjin43 cultivar (Zhang et al., 2022a). After NH_4_^+^ resupply, *CsAMT1.2*, *CsAMT2.2*, and *CsAMT2.3* genes were differentially induced in tea cultivars with different NH_4_^+^-uptake efficiency, indicating the uneven NH_4_^+^ transport capacity among cultivars (Zhang et al., 2018; Zhang et al., 2022b).

### 3.2 NO_3_^−^ transport

Membrane-bound nitrate transporters (NRTs) are required for NO_3_^−^ uptake in plants. The members of the large NRT family can be divided into four subfamilies: nitrate transporter 1/peptide transporter (NRT1/PTR), collectively known as NPF, nitrate transporter 2 (NRT2), chloride channel (CLC), and slow anion channel associated/homologue (SLAC/ SLAH) (Krapp et al., 2014).

The NRT1 subfamily harbors many members, acting in NO_3_^−^ transport from roots to shoots (Krapp et al., 2014). NRT transport activity is also regulated through phosphorylation. AtNRT1.1 is a dual-affinity protein: phosphorylation of the Thr101 residue by the CBL-interacting protein kinase 23 changes its substrate affinity (Sun et al., 2014). NRT2 are HATS proteins and belong to the nitrate/nitrite porter (NNP) family, mainly expressed in roots. These proteins have a role in both NO_3_^−^ accumulation and NO_3_^−^ transport (Chopin et al., 2007; Li et al., 2007; Kiba et al., 2012). To date, four *CsNRT1* and four *CsNRT2/3* genes have been isolated from tea plants. These genes show tissue-specific expression patterns and are differentially induced by exogenous NO_3_^−^. It was reported that *CsNRT1.1* and *CsNRT1.2* were mainly expressed in leaves. *CsNRT1.7*, *CsNRT2.5*, and *CsNRT3.2* had higher expression levels in mature leaves than other tissues, while *CsNRT1.5*, *CsNRT2.4*, and *CsNRT3.1* transcripts mainly accumulated in tea roots (Feng et al., 2014; Wang, 2014; Wang et al., 2014; Yang et al., 2016; Zhang et al., 2021). Further research showed that *CsNRT2.4* expression was root-specific and strongly induced by N resupply. *Arabidopsis* seedlings overexpressing *CsNRT2.4* had a significantly higher fresh weight and lateral roots length than wild-type seedlings, especially under low N availability (0.1 mM NO_3_^−^), pointing out CsNRT2.4 as a high-affinity nitrate transporter that might improve NO_3_^−^ uptake rate (Zhang et al., 2021). Additionally, Wang et al. (2022b) identified a total of 109 CsNPF members by analyzing the tea genome; these proteins could be divided into 8 groups according to their phylogenetic relationships, and the transcription of most of these genes responded to NO_3_^−^ supply. Similarly, *CsNRTs* expression profiles varied in tea cultivars with different NUE (Wang et al., 2014). The expression of *CsNRT2.4* and *CsNRT3.2* in the cultivar LongJin43 was higher than that observed in ZhongCha108, indicating higher responsiveness to external NO_3_^−^ supply in the former (Su et al., 2020).

Initially, CLC proteins were thought to be specifically involved in chloride (Cl^-^) transport as channels or 2 Cl^−^/1 H^+^ antiporters (Jentsch, 2008). Further research showed that AtCLCa is a tonoplast-located 2 NO_3_^−^/1 H^+^ antiporter that drives NO_3_^−^ accumulation in the vacuoles (Jentsch, 2008; Monachello et al., 2009). As anion channels, SLAC/SLAH proteins showed a strong preference for NO_3_^−^ and have been associated with CO_2_ and abscisic acid-dependent stomatal closure (Negi et al., 2008; Vahisalu et al., 2008). In tea plants, Xing et al. (2020) identified eight *CLC* genes across the wide genome of this species and named them *CsCLC1*-*8*. Phylogenetic studies demonstrated that the proteins encoded by these genes belonged to two subclasses; further studies showed that CsCLC transporters might participate in the uptake and long-distance transport of Cl^−^ and F^−^, as their expression levels varied in response to the addition of these two ions at different concentrations. However, the role of CsCLCs in NO_3_^−^ transport has not been elucidated. Similarly, there are no published reports related to SLAC/SLAH proteins in tea plants.

To summarize the precedent information, **Table 1** lists genes involved in NH_4_^+^ and NO_3_^−^ sensing, uptake, and transport in tea plants reported to date. Further information about the subcellular localization, sequence data, and functional corroboration experiments is also provided.

**Table 1:** Genes isolated from tea plants in NH_4_^+^ and NO_3_^−^ transport.

Gene name	Sequence information			Functional verification			References
	Gene ID ^a^		Cultivar ^b^	Subcellular localization	System ^c^	Function description	
*CsAMT1.1*	MV344632 KU361592		FD, LJ43	Predicted:plasma membrane	–	–	(Zhang et al., 2022a)
*CsAMT1.2*	MW344636 KU361593		FD,LJ43	Plasma membrane	Yeast *in planta* (*At.*)	A key gene for NH_4_^+^ uptake in roots	(Zhang et al., 2018; Zhang et al., 2022a; Zhang et al., 2022b)
*CsAMT1.3*	MW344633		FD	Predicted:plasma membrane	–	–	(Zhang et al., 2022a)
*CsAMT1.4*	MW344635		FD	Predicted:plasma membrane	–	–	(Zhang et al., 2022a)
*CsAMT1.5*	MW344634		FD	Predicted:plasma membrane	–	–	(Zhang et al., 2022a)
*CsAMT3.1*	KP338998		LJ43	Predicted:plasma membrane	–	–	(Zhang et al., 2018)
*CsAMT2.1*	MW751970		FD	Predicted:plasma membrane	Yeast	–	(Zhang et al., 2022b)
*CsAMT2.2*	MW751971		FD	Plasma membrane	Yeast	–	(Zhang et al., 2022b; Song et al., 2023)
*CsAMT2.3*	MW751972		FD	Plasma membrane	Yeast	–	(Zhang et al., 2022b; Song et al., 2023)
*CsAMT2.4*	MW751973		FD	Predicted:plasma membrane	Yeast	–	(Zhang et al., 2022b)
*CsAMT2.5*	MW751974		FD	Predicted:plasma membrane	Yeast	–	(Zhang et al., 2022b)
*CsNRT1.1*	–		ZC302	Predicted:plasma membrane	–	–	(Zhang et al., 2021)
*CsNRT1.2*	–		ZC302	Predicted:plasma membrane	–	–	(Zhang et al., 2021)
*CsNRT1.5*	–		ZC302	Predicted:plasma membrane	–	–	(Zhang et al., 2021)
*CsNRT1.7*	–		ZC302	Predicted:plasma membrane	–	–	(Zhang et al., 2021)
*CsNRT2.4*	–		ZC302	Plasma membrane	*in planta* (*Nt. At.*)	A key gene for NO_3_^-^ uptake in roots	(Zhang et al., 2021)
*CsNRT2.5*	–		ZC302	Predicted:plasma membrane	–	–	(Zhang et al., 2021)
*CsNRT3.1*	–		ZC302	Predicted:plasma membrane	–	–	(Zhang et al., 2021)
*CsNRT3.2*	–		ZC302	Predicted:plasma membrane	–	–	(Zhang et al., 2021)
*CsNPF2.3*	CSS0041711		ZM#6	Plasma membrane	*in planta* (*Nt.*)		(Wang et al., 2022b)
*CsNPF6.1*	CSS0037113		ZM#6	Plasma membrane			(Wang et al., 2022b)
*CsNRT*	KJ160503		–	–	–	–	(Wang, 2014)
*CsNRT1.2*	KP453862		LJ43	Predicted:plasma membrane	–	–	(Feng, 2014)

a Gene ID, the beginning as “CSS” can be found in the tea plant genome database (http://tpia.teaplants.cn), others are GenBank accession numbers (https://www.ncbi.nlm.nih.gov/genbank/);

b Cultivar, FD, FudingDaBaiCha; LJ43, LongJin43; ZC302, ZhongCha302; ZM#6, ZhongMing#6.

c System, *At*, *Arabidopsis thaliana*; *Nt*, *Nicotiana tabacum*.

-, related information not presented or studied in corresponding literature.

### 3.3 Amino acid- N transport

Tea plants can directly absorb organic N and transport it to actively growing parts. The amino acid theanine (Thea) is synthesized and stored in root cells and then transported from the root to the flush shoot in spring. These movements, which include xylem loading/unloading, xylem-to-phloem transfer, and post-vascular movements into the sink cells, are driven by plasmatic membrane-localized amino acid transporters (AATs) (Fischer et al., 1995; Dong et al., 2020; Lin et al., 2023a). Studies on tea plants AAT proteins have mostly focused on amino acid permeases (AAP), lysine and histidine transporters (LHT), and cationic amino acid transporters (CAT), which play important roles in AAs acquisition and long-distance transport from source to sink (Guo et al., 2019; Guo et al., 2020; Li et al., 2020; Liu, 2020).

Six CsAAPs members, CsAAP1, CsAAP2, CsAAP4, CsAAP5, CsAAP6, and CsAAP8, were identified in tea plants through the screening of a *Saccharomyces cerevisiae* mutant library. The expression of genes encoding these transporters was tissue-specific and regulated by the season and N levels. Thus, *CsAAP1* expression in roots increased in March and decreased by mid-April and was highly correlated with root-to-bud Thea transport in seven tea cultivars (Dong et al., 2020; Li et al., 2020). Besides, shading promoted *CsAAP2*, *CsAAP4*, *CsAAP5*, and *CsAAP8* expression in young stems and suppressed *CsAAP1*, *CsAAP2*, *CsAAP4*, *CsAAP5*, and *CsAAP6* expression in the leaves, in accordance with Thea levels in these tissues. These findings indicate that CsAAP2, CsAAP4, CsAAP5, and CsAAP8 functions may be related to Thea movements in the xylem, leading to high Thea accumulation in the stem. The up-regulated genes might induce Thea transport into the companion cells in the sieve elements for phloem loading and Thea delivery to the terminal leaves (Yang et al., 2021).

LHT proteins were investigated more deeply. The CsLHTs family comprises multiple members, among which CsLHT1 and CsLHT6, highly expressed in roots, were identified as H^+^-dependent high- and low-affinity amino acid transporters in yeast heterologous systems. The overexpression of *CsLHT1* and *CsLHT6* in *Arabidopsis* lines significantly increased the root ability to uptake exogenous nitrogen supplied as ^15^N-Gln and ^15^N-Glu, suggesting that these transporters may contribute to the use of organic N from the soil (Guo et al., 2019; Li et al., 2021). Likewise, the heterologous expression of *CsLHT4*, *CsLHT7*, and *CsLHT11* in *Arabidopsi*s was associated with a decline in aerial parts biomass compared with WT plants, but *CsLHT11* overexpressing plants had increased biomass in the rosette leaves, regardless the N levels. Therefore, this protein might have a regulatory function relevant to the development of harvestable, young shoots in tea plants (Huang et al., 2023b).

Regarding the cationic acid transporters, it was reported that the *CsCAT* gene family includes six members, mainly expressed in roots and stems. It was also found that some *CsCATs* modify their expression levels in response to abiotic stress and the exogenous application of Thea, Gln, and ethylamine hydrochloride, a precursor of Thea biosynthesis (Feng et al., 2018). CsCAT2 from tea plant was homologous to glutamine permease 1 (GNP1) from yeast, and it was found to be localized in the tonoplast as an H^+^-dependent amino acid transporter. *CsCAT2* was highly expressed in the roots in winter, and this was negatively correlated with Thea root-to-shoot translocation, providing evidence that this transporter may meditate Thea storage in tea cell vacuoles (Feng et al., 2021). These findings enrich our understanding of N homeostasis in the form of AAs. **Table 2** lists the genes involved in AAs transport in tea plants. When available, data on subcellular localization, sequencing, tea cultivars analyzed, specific substrates, and functional corroboration experiments are supplied.

**Table 2:** Genes isolated from tea plants in amino acids transport.

Gene name	Sequence information		Functional verification				Reference
	Gene ID ^a^	Cultivar ^b^	Subcellular localization	Substrate ^c^	System ^d^	Function description	
*CsAAP1*	TEA031577.1	SCZ	Plasma membrane and endoplasmic reticulum	Thea, Val, Asp, Glu, Gln, Ala, GABA	Yeast; *in planta* (*Nt. At.*)	Highly correlated to Thea root- to- shoot transport	(Dong et al., 2020; Li et al., 2020)
*CsAAP2*	TEA009392.1	SCZ	Plasma membrane and endoplasmic reticulum	Thea, Val, Asp, Glu, Gln, Ala, GABA	Yeast; *in planta* (*Nt. At.*)	–	(Dong et al., 2020; Li et al., 2020)
*CsAAP3*	TEA003112.1 MK532959	SCZ; LJ43	Predicted:plasma membrane	–	–	–	(Guo et al., 2020)
*CsAAP4*	TEA030129.1 MK532960	SCZ; LJ43	Predicted:plasma membrane	Thea, Val, Asp, Glu, Gln, Ala, GABA	Yeast	–	(Dong et al., 2020; Guo et al., 2020)
*CsAAP5*	TEA033139.1	SCZ	–	Thea, Val, Asp, Glu, Gln, Ala, GABA	Yeast	–	(Dong et al., 2020)
*CsAAP6*	TEA013446.1 MK532961	SCZ; LJ43	Plasma membrane and endoplasmic reticulum	Thea, Val, Asp, Glu, Gln, Ala, GABA	Yeast; *in planta* (*Nt. At.*)	–	(Dong et al., 2020; Guo et al., 2020; Li et al., 2020)
*CsAAP7*	TEA005296.1 MK532962	SCZ; LJ43	Predicted:plasma membrane	–	–	–	(Guo et al., 2020)
*CsAAP7.1*	XM_028244216.1	SCZ	–	–	–	–	(Li et al., 2022)
*CsAAP7.2*	MG523885	SCZ	Endoplasmic reticulum	Thea, Ala, GABA, Ser, Glu, Asn, Pro		Plays a role in AAs uptake from soil and Thea long- distance transport	(Li et al., 2022)
*CsAAP8*	TEA031424.1 MK532963	SCZ; LJ43	Predicted:plasma membrane	Thea, Val, Asp, Glu, Gln, Ala, GABA	Yeast	–	(Dong et al., 2020; Guo et al., 2020)
*CsAAP9*	TEA000756.1	SCZ	–			–	(Dong et al., 2020)
*CsLHT1*	TEA026462.1	SCZ; LJ43	Plasma membrane	Glu, Gln, Ala, Pro, Asn, Asp, GABA		H^+^-dependent high affinity transporter in uptake AAs from soil	(Guo et al., 2019; Li et al., 2021)
*CsLHT2*	TEA021847.1	SCZ	Predicted:plasma membrane	–	–	–	(Li et al., 2021)
*CsLHT3*	TEA033469.1	SCZ	Predicted:plasma membrane	–	–	–	(Li et al., 2021)
*CsLHT4*	TEA029168.1 CSS0010852.1	SCZ; FD	Predicted:plasma membrane	–	in planta (*At.*)	–	(Li et al., 2021; Huang et al., 2023b)
*CsLHT5*	TEA016092.1	SCZ	Predicted:plasma membrane	–	–	–	(Li et al., 2021)
*CsLHT6*	TEA003706.1	SCZ; LJ43	Plasma membrane	Glu, Gln, Ala, Pro, Asn, Asp, GABA		H^+^-dependent low affinity transporter in uptake AAs from soil	(Guo et al., 2019; Li et al., 2021)
*CsLHT7*	TEA021821.1 CSS0033052.1	SCZ; FD	Predicted:plasma membrane	–	in planta (*At.*)	–	(Feng et al., 2018; Li et al., 2021)
*CsLHT11*	CSS0019144.1	FD	–		in planta (*At.*)		(Huang et al. 2023b)
*CsLHT8.1*	–	LJ43	Predicted:plasma membrane	–	–	–	(Guo et al., 2019)
*CsLHT8.2*	–	LJ43	Predicted:plasma membrane	–	–	–	(Guo et al., 2019)
*CsCAT1*	KY709681	SCZ	–	–	–	–	(Feng, 2017; Feng et al., 2018)
*CsCAT2*	KY709679	SCZ	Tonoplast	Thea, Asp, Glu, Ala, Gln, Val		Meditate Thea storage	(Feng, 2017; Feng et al., 2018; Feng et al., 2021)
*CsCAT5*	KY709680	SCZ	–	–	–	–	(Feng, 2017; Feng et al., 2018)
*CsCAT6*	KY709682	SCZ	–	–	–	–	(Feng, 2017; Feng et al., 2018)
*CsCAT8*	KY709684	SCZ	–	Thea, Glu, Gln,	–	–	(Feng, 2017; Feng et al., 2018)
*CsCAT9*	KY709683	SCZ	–	Thea, Glu, Gln	–	–	(Feng, 2017; Feng et al., 2018)

a Gene ID, the beginning as “TEA” and “CSS” can be found in the tea plant genome database (http://tpia.teaplants.cn), others are GenBank accession numbers (https://www.ncbi.nlm.nih.gov/genbank/);

b Cultivar, SCZ, ShuChaZao; LJ43, LongJing43; FD, FudingDaBaiCha.

c Substrate, Thea, theanine; Glu, glutamate; Gln, glutamine; Asp, aspartate; Asn, Asparagine; Ala, alanine; Val, valine; Pro, proline; Ser, serine; GABA, γ-aminobutyric acid.

d System, *At*, *Arabidopsis thaliana*; *Nt*, *Nicotiana tabacum*.

-, related information not presented or studied in corresponding literature.

## 4 N utilization

### 4.1 NO_3_^−^ metabolic reduction

NO_3_^−^ absorbed by plants is a nitrogen form in a highly oxidized state, which must be reduced to NH_4_^+^ through metabolic reduction to be further utilized. In this process, nitrate reductase (NR) is the rate-limiting enzyme (Jackson et al., 2008). Both NR and NiR are substrate-inducible enzymes; their function is to transfer electrons for NO_3_^−^ reduction. NO_3_^−^ taken up by roots was reduced into ammonium in mesophyll cells of shoots, and the metabolic reduction can also be catalyzed in roots (Miller and Cramer, 2005). In rice, the alleles of *OsNR2* present differences between the two most common subspecies, *indica* and *japonica*. Thus, OsNR2 in *indica* rice promotes NO_3_^−^ uptake through OsNRT1.1B, conferring to this subspecies increased yield and greater NUE compared with *japonica* rice (Gao et al., 2019).

In tea plants, studies have mainly focused on the activity and expression of *CsNR* and *CsNiR*. The activity of NR was related to NO_3_^−^ content. Besides, this activity was lower in the less vigorously growing organs, such as the larger roots, older leaves and stems. In the new shoots, the *in vitro* NR activity decreased with the degree of leaf development, being highest in the first leaf and lowest in the fifth one (Wang and Su, 1990; Wu and Wu, 1993). NR activity was also responsive to trace elements including copper (Cu) and zinc (Zn). Foliar spraying of Cu and Zn increased the content of N-containing compounds and the activity of NR (Han and Wu, 1992). Under the same conditions, *CsNR* and *CsNiR* expression levels in tea roots were more strongly influenced by NH_4_^+^ than NO_3_^−^ (Tang et al., 2020). *CsNR* expression in tea roots was higher than in other tissues and was up-regulated by environmental stresses (Zhou, 2014). However, the expression level of this gene significantly differed across various cultivars (Zhou et al., 2013). On the other hand, the expression of *CsNiR* was higher in mature leaves than in new shoots and roots, and in roots, this gene expression was up-regulated after a short period of N resupply. The change in gene expression was slower in leaves, and this responsiveness also varied in different cultivars representing diverse genotypes (Zhang et al., 2016). Most of these findings correspond to earlier experiments; the experimental evidence for CsNR and CsNiR functions in tea plants is still scarce.

### 4.2 Ammonia assimilation

Both NH_4_^+^ absorbed directly by plant roots and NH_4_^+^/NH_3_ formed through NO_3_^-^ reduction can be derived to AAs synthesis using various keto acids generated through respiration; this process is known as ammonia assimilation. In higher plants, more than 95% of the NH_4_^+^/NH_3_ pool is assimilated via the GS-GOGAT cycle. Glutamine synthetase (GS) is the key enzyme in this pathway, playing a major role in fixing NH_4_^+^ to the δ-carboxyl group of Glu to form Gln (Thomsen et al., 2014). Tea plants have a particular ammonia-assimilation route; their roots can biosynthesize a unique amino acid, Theanine (Thea), a homolog of Gln (Lin et al., 2023a). Glutamate synthase (GOGAT) catalyzes the conversion of Gln and 2-oxoglutarate to Glu, thus providing Glu for ammonia assimilation (Bernard and Habash, 2009; Valderrama-Martín et al., 2022). When plants germinate, senesce, and begin to form seeds, glutamate dehydrogenase (GDH) can catalyze the reversible amination/deamination so that the GS-GOGAT cycle allows NH_3_ reuse, necessary for ammonia detoxification (Fontaine et al., 2012; Zhou et al., 2015). Through these pathways, N absorbed by roots is incorporated into proteins, nucleic acids, and other substances needed for plant growth.

#### 4.2.1 Glutamine synthetase (GS) and theanine synthetase (TS)

Two isoforms of GS were first identified by ion exchange chromatography: cytosolic GS (GS1) and plastidic GS (GS2) (Bernard and Habash, 2009). GS1 is localized in the cytoplasm of non-photosynthetic tissues and is mainly involved in assimilating NH_4_^+^ absorbed from the soil and released from the plant N cycle. GS2 is localized in the chloroplast stroma and is the main isoform in chlorenchyma, having a major role in NH_4_^+^ assimilation within the photorespiratory pathway and NO_3_^-^ reduction in plastids (Swarbreck et al., 2011; Thomsen et al., 2014).

GS has been studied in tea plants extensively. It may be noticed in the GenBank database that the Japanese researchers Tanaka and Taniguchi were the first to clone three CsGS1 genes from tea roots in 2011: *CsGS1.1* (AB115183), *CsGS1.2* (AB115184), and *CsGS1.3* (AB117934), but the functions of these genes had not been reported (Lin et al., 2023a). Tang et al. (2018) cloned three *CsGS1* genes from the leaf of the cultivar Longjing43; the information obtained from the sequence analysis showed that the 3’/5’-untranslated region differed from those obtained previously, and *CsGS1.1* and *CsGS1.3* were mainly expressed in roots, while *CsGS1.2* was mainly expressed in mature leaves. NH_4_^+^ or NO_3_^−^ supply also influences the expression levels of these genes. The expression of *CsGS1.1* in leaves was up-regulated only by NO_3_^−^ in a similar manner as *AtGLN1.2*, indicating that its role in ammonia assimilation originates from NO_3_^−^ reduction (Lothier et al., 2011; Guan et al., 2014). However, under the NH_4_^+^ treatment, the expression of *CsGS1.2* was induced in both leaves and roots, and *CsGS1.3* expression was only significantly increased in leaves (Tang et al., 2018). Further research indicated that GS activity in tea plants was quickly inhibited upon methionine sulfoximine addition, leading to the reprogramming of AAs and nitrogenated lipids. This change involved a decrease in the biosynthesis of all other AAs and nitrogenated lipids, whereas the content of NH_4_^+^, Thea, and glycolysis and tricarboxylic acid cycle-related metabolites increased, indicating that the inhibition reduced N reutilization in the leaves (Liu et al., 2019).

L-theanine (γ-glutamyl-L-ethylamide), also known as L-Thea, is a distinctive non-proteinogenic amino acid that contributes an umami taste and exhibits anti-depression benefits (Liu et al., 2017d). Thea accumulation was dynamically regulated by developmental growth, and environmental factors, including N supply, temperature, light intensity, and salt stress (Ashihara, 2015). The synthesis of L-Thea is a unique and highly characteristic aspect of nitrogen assimilation in tea plants. Deciphering the underlying molecular mechanism of L-Thea synthesis will provide valuable guidance for fertilization and breeding strategies. Theanine synthetase (TS), an essential enzyme for Thea metabolism, catalyzes the biosynthesis of Thea from ethylamine and Glu, mainly in tea roots (Fu et al., 2021a). The structure and properties of L-Thea are similar to those of L-Gln, and some studies have confirmed that TS is highly homologous to GS (Cheng et al., 2017). As indicated before, *CsTS1* (DD410895) and *CsTS2* (DD410896) were firstly isolated through cDNA library screening. *CsTS1* is mainly expressed in the new shoots, roots, and mature leaves, while *CsTS2* reached higher expression levels in shoots (Deng et al., 2008). Both genes are involved in Thea biosynthesis; this was validated through a heterologous expression system (Lin et al., 2023a).

By performing genome studies, Wei et al. (Wei et al., 2018) found that the predicted *CsGSⅠ* sequence shared high homology with that of *PtGS* (*Pseudomonas taetrolens*), and *PtGSI* has been engineered for Thea production at high levels, for which *CsGSⅠ* was renamed as *CsTSⅠ*. The function of CsTS and CsGS was investigated in depth through the transient overexpression in *Nicotiana benthamiana* leaves or the stable expression in *Arabidopsis* and knockdown in tea plants. The expression pattern and distribution of *CsTSⅠ* correlated with Thea and Gln contents in different tissues. CsTS I mainly accumulated in root tip epidermal, pericycle, and procambium cells to form cytoplasmic proteins. When fed with 10 mM ethylamine, *CsTSI*-overexpressing *Arabidopsis* seedlings showed a significantly higher Thea content than wild-type seedlings. Further research allowed the construction of *CsTSI* RNAi and *CsTSI* overexpressing chimerical tea seedlings with transgenic hair roots; the results demonstrated that the content of Thea decreased and that of Gln increased, thus proving that CsTSI biosynthesized Gln and Thea used glutamate as an acceptor and ammonium or ethylamine as a donor, respectively (Wei et al., 2018; Fu et al., 2021b; She et al., 2022). Fu et al. (2021b) used a non-aqueous fractionation method and could determine that, in roots, L-Thea biosynthesis mainly occurred in the cytosol through the action of the key and cytosolic enzyme L-Thea synthetase CsTSI, whereas in shoots, both the cytosol and chloroplasts were the major sites for L-Thea biosynthesis, and CsGS1.1 and CsGS2 were, most likely, the fundamental L-theanine synthetase. CsGS2 was identified as a key enzyme regulating Thea biosynthesis in chloroplasts, L-Thea content and distribution in leaf tissues would be affected by light, as long-term shading treatment led to a decrease in the proportion of L-Thea in the plastids by reducing *CsGS2* expression levels. Thus, new shoots could accumulate more L-Thea. In contrast, *CsGS1.2* expression in albino new shoots was higher than that found in common cultivars as a way to compensate for the low CsGS2 expression in undeveloped chloroplasts. These findings indicate that the mechanism underlying Thea synthesis might differ across tea genotypes (Yu et al., 2021).

#### 4.2.2 Glutamate synthase (GOGAT) and glutamate dehydrogenase (GDH)

There are two isoforms of GOGAT in plants, with different functions: ferredoxin-dependent GOGAT (Fd-GOGAT) and nicotinamide adenine dinucleotide-dependent GOGAT (NADH-GOGAT). Fd-GOGAT assimilates ammonia through photorespiration in leaves, while NADH-GOGAT accumulates in non-green tissues, playing a role in ammonia assimilation in root (Suzuki and Knaff, 2005; Konishi et al., 2014).

GDH is abundant in plant tissues; this enzyme catalyzes ammonia conversion to Glu and also deaminates Glu to α-ketoglutarate. GDH-mediated ammonia assimilation and as a stress-responsive enzyme, GDH detoxified the intracellular high ammonia and biosynthesize Glu (Lea and Miflin, 2003; Fontaine et al., 2012; Zhou et al., 2015). *CsGOGAT* was found to have significantly higher expression in the leaf than in the root (Chen et al., 2015). Under N starvation, *CsGOGAT* expression increased, and *CsGDH* expression decreased significantly; these changes were correlated with leaf N content (Lin et al., 2014). CsGOGAT also have a regulatory role in AAs changes in postharvest tea plant leaves. The Thea content changed in spreading tea leaves under different treatments, and CsGOGAT was involved in Thea metabolic pathway, regardless of external light and temperature. Also, CsGOGAT would interact with CsTS I and CsNiR during N metabolism (Liu et al., 2017c). In tea plant, all CsGDHs identified to date belong to the NADH-GDH group. Accumulation of *CsGDH2* transcripts seemed to be flower-specific compared with the other five plant tissues analyzed; *CsGDH1* was mainly expressed in mature leaves and roots, and *CsGDH3* in new shoots and roots. Under high NH_4_^+^ supply, CsGS inhibition resulted in a significant up-regulation of *CsGDH3* and *CsGDH2* in roots and leaves, indicating the synergistic effect of CsGSs and CsGDHs in the process of ammonia assimilation (Tang et al., 2021). The expression of *CsGDH2.1* in shoots increased greatly in the late spring; further investigation revealed that Glu was a signal for Thea hydrolysis, and CsGDH2.1-mediated Glu catabolism negatively regulated Thea accumulation in the new shoots in the late spring, improving green quality by targeting to reduce *CsGDH2.1* expression (Chen et al., 2022).

Summing up, studies directed to analyze the genes related to N metabolism in tea plants mostly focused on their function in regulating AAs biosynthesis. Knowledge about the functioning and regulation of the enzymes involved in these processes is mostly based on transcript analyses. There are still many gaps in our understanding of their functions, especially for NR, NiR, and GOGAT, concerning NO_3_^−^ reduction, N assimilation, remobilization, and reassimilation of photorespiratory NH_3_. It is noteworthy that, apart from the transcriptional regulation, post-translational modifications (PTMs) can also be critical for the regulation of many proteins relevant to N metabolism in plants (Liu et al., 2022). Therefore, more detailed studies will deepen our understanding of NUE determinants and allow further optimization of NUE under actual tea garden production scenarios.

Genes involved in ammonia assimilation by tea plants and their most relevant data are shown in **Table 3**.

**Table 3:** Genes involved in ammonia assimilation in tea plant.

Gene name	Sequence information		Functional verification			References
	Gene ID ^a^	Cultivar ^b^	Subcellular localization	System ^c^	Functional description	
*CsNR*	JX987133	LJ43	–	–	–	(Zhou et al., 2013)
*CsNiR*	–	LJ43	–	–	–	(Zhang et al., 2016)
*CsGS1.1*	AB115183 KY649469 TEA015580.1 MG778703	‘Sayamakaori’ posterity LJ43 JX	Cytosol and nucleus	*E. coli* *in planta* (*At.*)	Biosynthesizes Thea and Gln	(Cheng et al., 2017; Tang et al., 2018; Wei et al., 2018; Fu et al., 2021b; Yu et al., 2021)
*CsGS1.2*	AB115184 KY649470 TEA032123.1 MG778705	‘Sayamakaori’ posterity LJ43; JX	Cytosol	*E. coli* *in planta* (*At.*)		
*CsGS1.3*	AB117934 KY649471 TEA032217.1 MG778704	‘Sayamakaori’ posterity LJ43; JX	Mitochondria	*E. coli* *in planta* (*At.*)		
*CsGS2*	TEA028194.1 MG778706	JX	Chloroplast, mitochondria	*E. coli* *in planta* (*At.*)	Thea synthetase in chloroplasts	(Cheng et al., 2017; Wei et al., 2018; Fu et al., 2021b; Yu et al., 2021)
*CsGS*	EF055882	–	–	–	–	(Rana et al., 2008)
*CsGS*	JN602372	JLP	–	–	–	(Lin et al., 2014)
*CsTSⅠ*	TEA015198.1	SCZ	Cytosol	*in planta* (*At.* Tea plant hairy roots)	Thea synthetase in cytosol.	(Wei et al., 2018; Fu et al., 2021b)
*CsTS1*	DD410896	–	–	*E. coli*	Biosynthesizes Thea after supply with ethylamine	(Cheng et al., 2017; Fu et al., 2021b)
*CsTS2*	DD410895	–	–	*E. coli*		(Cheng et al., 2017; Fu et al., 2021b)
*CsTS3*	JN226569	AJB	Predicted: cytoplasm peroxisome	–	–	(Li et al., 2011; Chen et al., 2015)
*CsGOGAT*	JN602373	JLP	–	–	–	(Lin et al., 2014)
*CsGOGAT1*	TEA003892.1	**–**	**–**	**–**	**–**	(Wei et al., 2018; Li et al., 2019)
*CsGOGAT2*	TEA026779.1	**–**	**–**	**–**	**–**	(Wei et al., 2018; Li et al., 2019)
*CsGOGAT3*	TEA030315.1	**–**	**–**	**–**	**–**	(Li et al., 2019)
*CsFd–GOGAT*	–	LJ43	**–**	**–**	**–**	(Liu et al., 2017c)
*CsNADH–GOGAT*	–	LJ43	**–**	**–**	**–**	(Liu et al., 2017c)
*CsGDH*	JN602371	JLP	**–**	**–**	**–**	(Lin et al., 2014)
*CsGDH1*	TEA034004.1	LJ43	**–**	**–**	**–**	(Tang et al., 2021)
*CsGDH2*	TEA009809.1	LJ43	**–**	**–**	**–**	(Tang et al., 2021)
*CsGDH3*	TEA034006.1 TEA006665.1	LJ43	**–**	**–**	**–**	(Tang et al., 2021)
*CsGDH2.1*	CSS0034454.1	SCZ	Mitochondria	Yeast *in planta (Nt.)* asODN in tea plant	Negatively regulates theanine accumulation in the late–spring	(Chen et al., 2022)
*CsGDH2.2*	CSS0007238.1	SCZ	Mitochondria	Yeast *in planta (Nt.)*	**–**	(Chen et al., 2022)

a Gene ID, the beginning as “TEA” and “CSS” can be found in the tea plant genome database (http://tpia.teaplants.cn), others are GenBank accession numbers (https://www.ncbi.nlm.nih.gov/genbank/);

b Cultivar, LJ43, LongJing43; JX, JinXuan; SCZ, ShuChaZao; JLP, JiuLongPao; AJB, AnJiBaicCha.

c System, *E. coli*, *Escherichia coli*; *At*, *Arabidopsis thaliana*; *Nt*, *Nicotiana tabacum*; asODN, antisense oligonucleotide.

-, related information not presented or studied in corresponding literature.

## 5 An overview of nitrogen use efficiency assessment by tea plants

Nitrogen use efficiency (NUE) is a complex trait influenced by the interaction between environmental factors and intrinsic plant factors; this variable can be approached at different levels and calculated in different ways (Santa-María et al., 2015). Initially, NUE was defined as the crop yield per unit of applied N, a parameter also termed partial fertilizer productivity (PFP) (Moll et al., 1982). Under specific N supply conditions, NUE can be divided into two components: nitrogen uptake efficiency (NUpE) and nitrogen utilization efficiency (NUtE) or nitrogen physiological efficiency (NPE). NUpE may be defined as the total amount N absorbed and NUtE as the dry weight or grain yield per unit of absorbed N, accounting for the results at this growth stage (Williams et al., 2021). Tea germplasm resources are abundant in China; the genetic diversity of this plant, resulting from a long time of artificial domestication and cultivar-breeding improvement, has determined quite different N requirements (Zhang et al., 2018). Additionally, because tea production does not target grain yield, dissimilar NUE assessment criteria were adopted. Here, we integrate the results of several studies and present four approaches to assess NUE by tea plants.

### 5.1 Biomass accumulation

By the end of the 20^th^ century, it was reported that the rate of increase in tea ground stem diameter and height and dry matter production in different cultivars varied under sufficient N supply compared to no N application (Ruan et al., 1993). Under low N supply, tea plants’ height, root and shoot dry weight, and leaf SPAD values were significantly decreased (Wang et al., 2015). Wang et al. (2004) measured the added-N content in the biomass and the growth of new shoots in six tea cultivars under four N levels (based on ^15^N isotope labeling techniques), and redefined five interdependent traits—nitrogen use efficiency (NE), nitrogen uptake efficiency (NUE), nitrogen physiological utilization efficiency (NPE), nitrogen economic efficiency (NEE) and N responsiveness—according to growth characters and harvesting organs. They found that the biomass increase was significantly correlated with NEE, the weight of the new shoots was significantly (positively) correlated with NE, NUE, and NEE, while NUE was the main determinant of NE. These authors indicated that by comparing the NE values of different cultivars, it is possible to detect which cultivar can achieve the highest NUE for a given level of N supply.

### 5.2 Root-related traits

The root is the main organ for nutrient uptake and plays a direct role in N acquisition (Lynch, 2007; Zhu et al., 2011). Root development and activity are responsive to soil N levels (Ju et al., 2015). Studies on plant response to N concentration gradients using different tea cultivars suggested that N concentration has a significant effect on root/shoot ratio, and this ratio could be used as a screening index to detect low-N-tolerant cultivars (Wang et al., 2015). On the other hand, the differences among cultivars in root-related parameters such as root dry weight, root volume, or root active uptake area were greater than those of root activity. Likewise, root volume and active uptake area varied significantly across N levels. Further correlation studies provided evidence that these parameters may be considered as promising indices for selecting and breeding tea cultivars with high NUE (Wang et al., 2005).

### 5.3 NH_4_^+^ influx kinetics

In the early 1950s, Epstein and Hagen (1952) applied the Michaelis-Menten equation for the first time to describe the absorption process of ionic nutrients by plants. In this equation, V_max_ represents the maximum uptake rate; this value is directly proportional to the uptake rate for ions, and K_m_ is inversely proportional to the affinity of the cell membrane for nutrient ions (Zhang et al., 2018). Because tea roots show a preference for NH_4_^+^ uptake as the nitrogen source, the kinetic parameters of this cation are usually used to define tea adaptability to N availability. According to current studies on NH_4_^+^ dynamics, tea cultivars may be classified into three categories: (1) cultivars with high K_m_ and high V_max_ can produce high yields in soils with elevated N contents; TeiGuanYin, HuangDan, and Yubukita cultivars belong to this type; (2) cultivars with low K_m_ and low V_max_ may display a good performance in soils with low N concentrations; YingShuang and MaoXie belong to this type; (3) cultivars with high K_m_ and low V_max_ are the most flexible concerning N levels, being appropriate for both high and low N conditions; ZhongCha#302 and FuDingDaBaiCha belong to this type (Wang et al., 2005; Liu, 2016; Zhang et al., 2018; Zhang et al., 2022b). Notably, N flux was calculated as the N content in the roots based on ^15^N labeling in most studies, and there are still many cultivars falling into different groups in different studies due to different number of tested cultivars and methodological approaches. Though NH_4_^+^ influx kinetics allowed a better understanding of N use by tea plants, more precise methods, such as non-invasive procedures based on micro-test technology, will be useful for future experiments (Ruan et al., 2016; Su et al., 2020).

### 5.4 Activity and gene expression of N-assimilation-related enzymes

The leaves are the main assimilation organs of inorganic N. The accumulation of N-assimilates and the enzymes and genes regulating these metabolic processes could indicate NUE-related sub-traits (Sun et al., 2019). Some studies showed that GS activity varied among cultivars and N levels and was positively correlated with N assimilation rate and NUpE (Wang et al., 2005; Du et al., 2015). Lin et al. (2017; 2018) examined the activity of some antioxidant enzymes and found increased activities in the low N-tolerant cultivar HuangDan in a nitrogen-deficient environment. This was linked to the maintenance of high photosynthetic rates and to the adequate output of N-assimilates in the leaves. Still, by combining genes, enzymes, and assimilates and exploring their affiliation links, it was possible to evaluate NUE traits comprehensively. Zhou (2012) measured soluble sugars, soluble proteins, total N content, N-related enzymatic activities, and the expression of AAs biosynthetic genes. Their results suggested that the differences in these indicators varied in the five cultivars tested as the N concentration increased and membership function could be used to evaluated the NUE of each cultivar synthetically. Also, *CsAMTs* expression profiles in response to NH_4_^+^ differed among cultivars (Zhang et al., 2018; Zhang et al., 2022b). Still, it was possible to detect that CsNRT2 participated in NO_3_^−^ transport under low N conditions (Hu et al., 2023; Lin et al., 2023b). The AuTophaGy-related genes *CsATG8e* and *CsATG3a* were linked to an improved plant ability for N recycling and tolerance to low N levels (Huang et al., 2020; Huang et al., 2023a). These genes emerge as promising indicators and may contribute to identifying higher NUE among various germplasm resources.

Although multiple investigations have addressed NUE of tea plants, most NUE-related traits were identified based on individual morphology, physiological processes, relevant biochemical components, or gene expression patterns. Nevertheless, there are no universal standards for grading NUE in tea plants, and some cultivars have shown heterogeneous results. The measurement of biomass is time-consuming and susceptible to environmental changes. And only the processes of N uptake, transport or utilization not the comprehensive NUE have been measured in tea plants. The practicability of method also depends on the number of tested cultivars. Most importantly, NUE estimates are complicated and current evaluation methods are not comprehensive enough to cover and explain the meaning of NUE. The methodological limitations still resist our understanding of N metabolic mechanisms. Therefore, analyses combining omics data and molecular and genetic approaches will be useful to elucidate further heritability and inheritance in this species with a point of great value to improve NUE by tea plants.

## 6 Conclusions and perspectives

N is the driving factor for tea yield and quality. Facing the practical problem of the disproportionate amount of N fertilizers applied and the low N utilization rate by tea plantations, a comprehensive study on the process of N transport, absorption, and utilization is necessary to increase NUE, to improve quality features such as aroma and flavor, and, ultimately, to promote the sustainable development of the industry.

Currently, it is clear that tea plants show a preferential uptake and assimilation of NH_4_^+^ over NO_3_^−^, and more NH_4_^+^ availability allows tea plants to produce more AAs, which further act as signaling molecules involved in other metabolic pathways. In addition, great progress has been made in the elucidation of the N primary metabolism network. Genes contributing to N transport and assimilation have been cloned and sequenced, and the functions of many genes have been identified by transgenic experiments in yeast, *Arabidopsis*, and *Nicotiana tabacum*. However, the current methods to assess tea NUE under actual productive settings have limitations. For instance, some basic indices related to plant physiological performance and gene expression were proposed, but these approaches are time-consuming and inappropriate for large-scale field cultivar assessment. One drawback is that a stable transgenic system has not been established yet; hence, we cannot knock out or overexpress genes to provide functional evidence in homologous systems. Therefore, there is an urgent need to develop an efficient and stable gene transformation system for tea plants, even more considering that the N metabolism network is regulated by multiple genes. Future research should consider the following issues.

Firstly, most research on N uptake and utilization by tea plants has focused on ammonia assimilation and AAs biosynthesis. However, NH_4_^+^-based fertilizers and urea are widely applied in tea gardens, and these N forms are expected to be converted to NO_3_^−^ by nitrification, entailing the risk of leaching. It has been reported that NO_3_^-^ was the main chemical form of N loss by leaching: about 51%-63% of the added N is lost in this way (Zheng, 2022). Therefore, the biological significance of NRT, NR, and NiR in N utilization is not negligible. In rice, the nitrate sensor NRT1.1B could perceive NO_3_^−^ signal at the plasma membrane and facilitated SPX4 degradation by recruiting NBIP1, resulting in the cytoplasm-to-nuclear shuttling of OsNLP3 to transduce NO_3_^−^ response (Hu et al., 2019). Also, in *Arabidopsis*, the phosphorylation state of NRT1.1 regulates the nitrate signaling for lateral root growth, and the non-phosphorylable NRT1.1^T101A^ would activate Ca^2+^-CPKs-NLPs signaling pathway by inducing its endocytosis under high NO_3_^−^ concentration (Zhang et al., 2019a).

Secondly, although significant progress has been made in recent years regarding our understanding of the transcriptional regulation of the GS-GOGAT cycle, there are few reports on how transcription factors (TFs) regulate the expression of these downstream genes. The latest research revealed that the lateral organ boundaries domain gene *CsLBD39* negatively regulated NO_3_^−^ transduction (Teng et al., 2022). Functional studies on the regulation of N metabolism by TFs need to be further expanded in both scope and depth. Additionally, PTMs also influence NUE through their effects on relevant proteins in plants. Phosphorylation and dephosphorylation of NR are involved in regulating NR activity, and phosphorylation, oxidation, tyrosine nitration, and S-nitrosylation of GS protein are also key mechanisms for GS function in many crops, including wheat, rice, and maize (Liu et al., 2022). A recent study in tea plants found that CsALT, CsTSI, CsGS, and CsAlaDC, proteins involved in Thea synthesis, were modified through ubiquitination, implying that these enzymes’ stabilities were regulated by this modification (Wang et al., 2021b). Consequently, to establish a comprehensive N mechanism network for tea plant, N transport, reduction and assimilation requires precise regulation at both the transcriptional and post translational levels, many efforts need to be made to explore the PTMs, particularly to identify the modification sites that may be relevant for N use regulation by tea plants.

Furthermore, plants can respond to changes in N uptake by adjusting leaf expansion and photosynthetic rates, as well as chlorophyll content. In senescent leaves, N assimilation decreased; this was associated with the degradation of proteins and nucleic acids; the released N was remobilized to developing tissues. The expression of genes related to GS/GOGAT cycle during leaf senescence was widely investigated; most of these genes were expressed in phloem companion and parenchyma cells in cereals, suggesting that GS/GOGAT cycle plays a vital role in N remobilization from senescent organs to developing organs (Havé et al., 2017; Liu et al., 2022). In addition, NH_4_^+^, NO_3_^−^, AAs, and peptide transporters also can be up- or down-regulated during leaf senescence. Thus, many aspects of N metabolic pathways would be influenced by N recycling and remobilization (Breeze et al., 2011). It is reasonable to hypothesize that there are some other undiscovered factors and pathways, for example, components of C metabolism that regulate N remobilization. Tea production involves the pruning and picking of the tender leaves every season; this leads to a more active N turnover between the senescent leaves and the new shoots. Hence, for tea plants, an overview of the mechanisms involved in N recycling and remobilization is important to improve N resorption efficiency and also to reduce the use of N chemical fertilizers, which are responsible for a large part of greenhouse gas emissions.

Finally, along with the deciphering of the tea genome in multiple cultivars (Wang et al., 2021a), whole genome resequencing could provide more efficient single nucleotide polymorphisms (SNPs) markers to construct a high-density linkage map of tea populations. Such maps will lay a foundation for further investigations of quantitative trait loci (QTL) mapping and genome-wide association studies (GWAS) in order to reveal the molecular basis for important agronomic traits. In rice, forward genetics approaches revealed that allelic variation at *OsNR2* and *OsNRT1.1B* resulted in the nitrate-use divergence between *indica* and *japonica* subspecies and were used to improve the NUE of rice (Hu et al., 2015; Gao et al., 2019). Multiple attempts have been made to detect relevant QTLs or variation sites and quality-related traits in tea plants, including biochemical components, leaf area (An et al., 2021), seed setting rate (Wei et al., 2021), bud flush timing (Tan et al., 2022), and AAs (Huang et al., 2022), caffeine (Ma et al., 2018), and flavonoid (Xu et al., 2018) contents. However, fewer attempts to unravel nutrient uptake and utilization traits in the context of genotype-to-phenotype mapping research have been reported. Nutrient-related traits are generally regulated by multiple genes and environmental factors, so it is difficult to quantify their phenotypes precisely. More attempts need to reveal the processes of N cycling, and to define the phenotypic indicators that reflect each step of N metabolism. For example, chlorate (ClO_3_^−^) is an analogic tracer for NO_3_^−^ and the resistance ability to ClO_3_^−^ is an efficient indicator for fast screening the process of NO_3_^−^ transport and reduction divergency (Hu et al., 2015). How to apply this method in woody plants is a challenge that needs to be considered in future research. Population genetics can help us to explore better the gene regulatory loci affecting NUE-related traits and to identify the TFs or promoters which are able to regulate or activate the transcription of downstream structural genes. Exploiting interpopulation genetic variation in different germplasms will be instrumental for cultivar improvement. Thus, the use of precise phenotyping methods on population is challenging but necessary for future studies of discovering genetic variation associated with NUE-related traits.

Therefore, future studies should focus on the regulation mechanisms of NO_3_^−^ uptake and reduction in tea plants to increase the utilization of NO_3_^−^ from the soils and reduce leaching losses, a point of great significance for the genetic improvement directed to high NUE cultivars as well as for developing a sustainable tea plantations.

## Author contributions

WZ and LL contributed to the conceptualization. WZ prepared the first draft and figures. LL and KN contributed with inputs and made revisions in the text and figures. LL and JR supervised the overall process. All authors contributed to the article and approved the submitted version.
